# Supplementary material for: Epidemiology and burden of respiratory syncytial virus in Italian adults: A systematic review and meta-analysis
Source: PLoS One. 2024 Mar 5;19(3):e0297608. doi: 10.1371/journal.pone.0297608 (PMC10914269; doi:10.1371/journal.pone.0297608)

**S1 Fig.** RSV positivity prevalence among Italian adults of any age, by study period in relation to the COVID-19 pandemic.


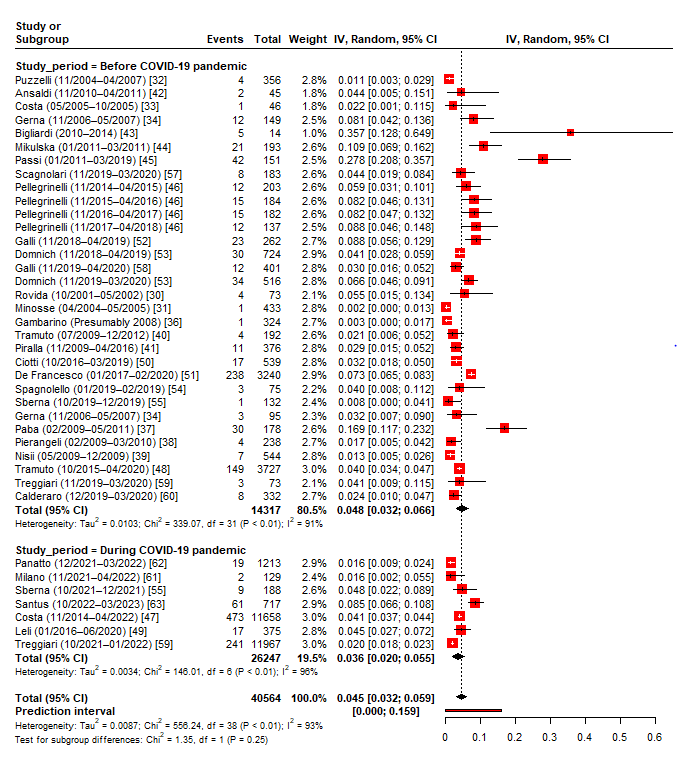

Supplement: S1 Fig — (DOCX) [file pone.0297608.s001.docx]
