# Supplementary figures and images for: Epidemiology and burden of respiratory syncytial virus in Italian adults: A systematic review and meta-analysis
Source: PLoS One. 2024 Mar 5;19(3):e0297608. doi: 10.1371/journal.pone.0297608 (PMC10914269; doi:10.1371/journal.pone.0297608)

**S2 Fig.** RSV positivity prevalence among Italian adults of any age, by study geographic area.


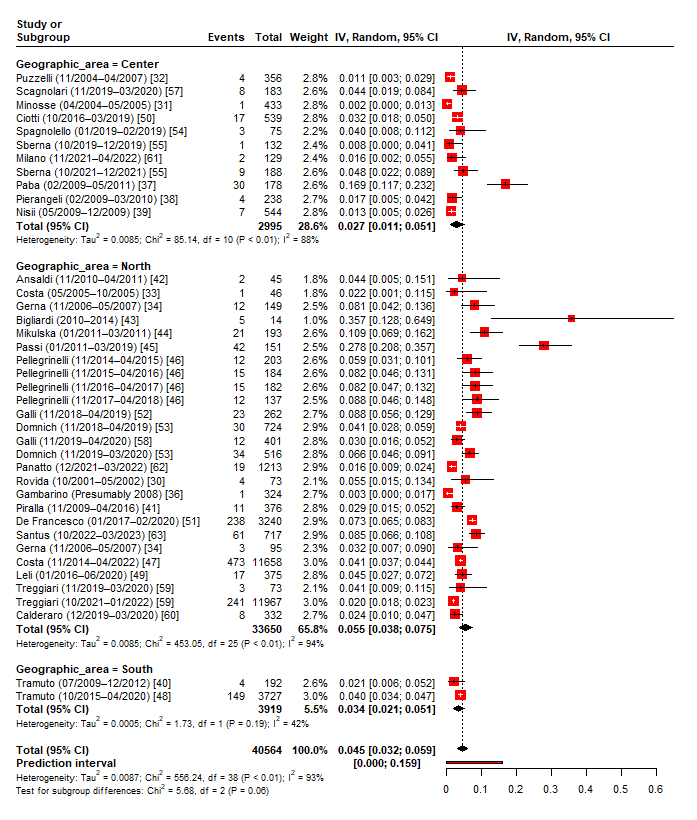

Supplement: S2 Fig — (DOCX) [file pone.0297608.s002.docx]

**S3 Fig.** RSV positivity prevalence among Italian adults of any age, by study sample size.


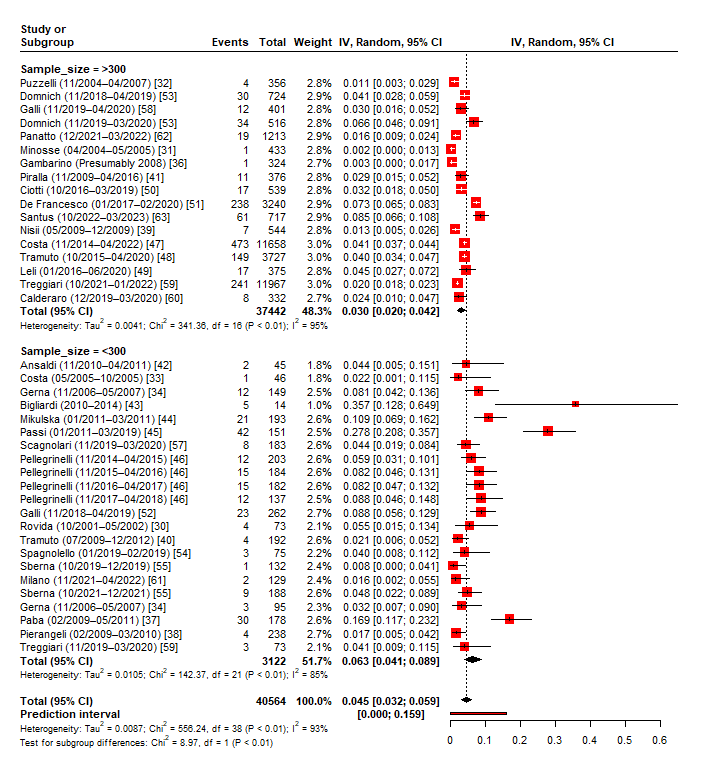

Supplement: S3 Fig — (DOCX) [file pone.0297608.s003.docx]

**S5 Fig.** Frequency of viral co-detections among RSV-positive Italian adults of any age.


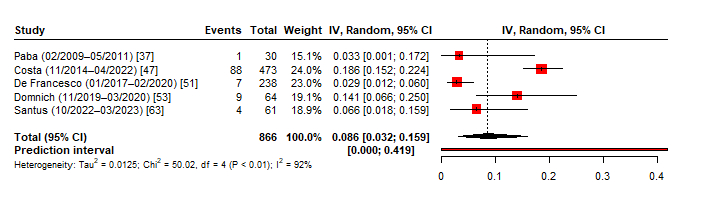

Supplement: S5 Fig — (DOCX) [file pone.0297608.s005.docx]

**S6 Fig.** In-hospital mortality among RSV-positive Italian adults of any age.


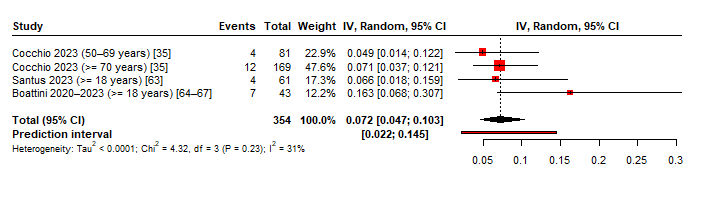

Supplement: S6 Fig — (DOCX) [file pone.0297608.s006.docx]
