# Supplementary material for: Epidemiology and burden of respiratory syncytial virus in Italian adults: A systematic review and meta-analysis
Source: PLoS One. 2024 Mar 5;19(3):e0297608. doi: 10.1371/journal.pone.0297608 (PMC10914269; doi:10.1371/journal.pone.0297608)

**S4 Fig.** Prevalence of RSV subtype B among Italian adults of any age (prevalence of RSV subtype A may be computed as 1 – prevalence of RSV B).


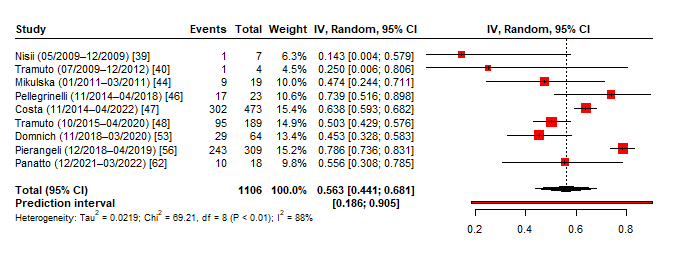

Supplement: S4 Fig — (DOCX) [file pone.0297608.s004.docx]
