# Supplementary material for: Epidemiology and burden of respiratory syncytial virus in Italian adults: A systematic review and meta-analysis
Source: PLoS One. 2024 Mar 5;19(3):e0297608. doi: 10.1371/journal.pone.0297608 (PMC10914269; doi:10.1371/journal.pone.0297608)
Supplement: S2 Table — (DOCX) [file pone.0297608.s008.docx]

**S2 Table.** Algorithm for the automatic search (last search performed on 22 November 2023), by citation database.

| **Database** | **Script** | **Records retrieved** |
| --- | --- | --- |
| MEDLINE, Biological Abstracts and Global Health via Ovid | 1 (respiratory syncytial or rsv).mp | 38160 |
|  | 2 exp Respiratory Syncytial Viruses/ or exp Respiratory Syncytial Virus, Human/ or exp Respiratory Syncytial Virus Infections/ | 15422 |
|  | 3 1 or 2 | 38160 |
|  | 4 exp Adult/ or exp Aging/ | 10588199 |
|  | 5 Men/ | 4481 |
|  | 6 Women/ | 16721 |
|  | 7 Retirement/ | 12457 |
|  | 8 ((old* or age*) adj3 (people* or person* or adult* or women* or men* or citizen* or residen*)).tw. | 1713324 |
|  | 9 (pension* or retire* or adult* or aged or elderly or senior* or geriatric*).tw. | 5242094 |
|  | 10 long-term care/ or nursing care/ or palliative care/ | 135907 |
|  | 11 homes for the aged/ or nursing homes/ | 49324 |
|  | 12 nursing home*.tw. | 46295 |
|  | 13 or/4-12 | 12562758 |
|  | 14 exp Italy/ or (Italy or Italian*).mp or (Italy or Italian*).tw | 335766 |
|  | 15 3 and 13 and 14 | 135 |
|  | 16 Remove duplicates from 15 | 84 |
| Scopus | TITLE-ABS-KEY ({respiratory syncytial} OR rsv*) AND ALL (adult* OR elder* OR older*) AND TITLE-ABS-KEY (Italy OR Italian*) | 119 |
| Web of Science | 1 TS=(respiratory syncytial* or rsv*) | 31474 |
|  | 2 TS=(Italy* or Italian*) | 364382 |
|  | 3 TS=(adult* or aged or elderly or senior* or geriatric* or retire* or pension* or old* people or old* person* or old* adult* or old* men or old* women or old* citizen* or old* residen* or nursing home*) | 5970623 |
|  | 4 #1 AND #2 AND #3 | 109 |
